# CC12 Induces Apoptotic Cell Death and Cell Cycle Arrest in Human Glioblastoma Cell Lines and Mouse Xenograft Model

**DOI:** 10.3390/molecules25081793

**Published:** 2020-04-14

**Authors:** Li-Yun Fann, Jui-Hu Shih, Jen-Ho Tseng, Hsu-Shan Huang, Sheng-Huang Hsiao

**Affiliations:** 1 Department of Nursing, Taipei City Hospital, Taipei 10684, Taiwan; fanly99@gmail.com; 2 Department of Biology and Anatomy, National Defense Medical Center, Taipei 11490, Taiwan; 3 Department of Pharmacy Practice, Tri-Service General Hospital, Taipei 11490, Taiwan; jtlovehl@gmail.com; 4 School of Pharmacy, National Defense Medical Center, Taipei 10684, Taiwan; 5 Department of Neurosurgery, Taipei City Hospital, Taipei 10684, Taiwan; DAL87@tpech.gov.tw; 6 Graduate Institute for Cancer Biology & Drug Discovery, College of Medical Science and Technology, Taipei Medical University, Taipei 11064, Taiwan

**Keywords:** apoptosis, brain tumor, cell arrest, chemotherapy, DNA damage, glioblastoma

## Abstract

Among central nervous system tumors, glioblastoma (GBM) is the most common and the most malignant type. Even under current standard treatments, the overall survival rate is still low and the recurrence rate is high. Therefore, developing novel and effective therapy is urgently needed. CC12, a synthesized small molecule, was evaluated for the potential anti-GBM effects in two GBM cell lines, U87MG and U118MG. The observations of cell morphology, MTT assay, flow cytometry-based apoptosis after CC12 treatment, were conducted. Western blot was performed for the investigation of the apoptotic mechanism. Positron emission tomography scan analysis and bioluminescent imaging assay using a mouse xenograft model were performed for the effect of CC12 in vivo. After treated by 10 μM CC12 for 24 h, both U118MG and U87MG cells showed tumor cell death. MTT assay results showed that the survival rates decreased when the CC12 concentrations or the treatment periods increased. Ki-67 expression and flow cytometry results indicated that the proliferation was inhibited in GBM cells, and G1 phase arrest was shown. The results of 7-AAD, Br-dUTP, and JC-1 staining all showed the apoptosis of GBM cells after CC12 treatment. Increased γH2AX, caspase-3, and poly (ADP-ribose) polymerase (PARP) levels meant the DNA damage, and increased Bcl2 family proteins after CC12 treatment indicated the intrinsic apoptotic pathway was involved in CC12 induced apoptosis. Furthermore, CC12 can induce the decrease of tumor prognostic marker DcR3. In vivo experiment results showed the effect of CC12 on tumor size reduction of CC12. In addition, the ability to cross the brain–blood barrier of CC12 was also confirmed. CC12 may have anti-tumor ability through the regulation of cell cycle and apoptosis in vitro and in vivo.

## 1. Introduction

Primary central nervous system (CNS) tumors are a group of malignancies with diversely histological characteristics. In the World Health Organization (WHO) classification system, over one hundred distinct entities were identified [1]. Gliomas is one of the major types of CNS tumors and is classified to several subtypes, including oligodendroglioma, oligoastrocytoma, astrocytoma, and glioblastoma (GBM), which is the most malignant astrocytic tumor with WHO grade IV, characterized by poorly differentiated neoplastic astrocytes with cellular polymorphism, nuclear atypia, high mitotic activity, necrosis, vascular proliferation, and thrombosis [1]. GBM is the most common type of CNS tumor in adults, accounting for 46.6% of all brain tumors; the incidence of GBM increases with age, with a median age of 64 years at diagnosis [2]. GBM is very aggressive, even under the current standard of treatment, the medium overall survival from diagnosis is around 15 months, the 2-year survival rate is 26–33%, and the 5-year survival rate is only 5.5% [2,3].

The current treatment of GBM consists of surgical resection, radiation, and chemotherapy. The critical therapeutic strategy is still the maximal safe surgical resection [4]. However, since the invasiveness of GBM, the tumor may invade through the brain parenchyma, subarachnoid space, perivascular space, and white matter tracts [5]; thus, complete and safe resection may not be achieved in some patients. Accompanied by post-operative radiation and concomitant, maintenance chemotherapy is necessary in most patients to prevent tumor recurrence. Carmustine, lomustine, fotemustine, and temozolomide are used for GBM chemotherapies; the mechanism, mainly, is inducing cell death or inhibiting cell proliferation. These drugs should be able to cross the brain–blood barrier (BBB) and achieve therapeutic concentrations in the CNS; thus, they usually have a small molecule weight and high lipophilicity. However, resistance to the drugs has been reported frequently [6]. Despite the prognosis for patients with GBM being poor, and the reported resistances of currently used chemotherapy [6], the development of novel effective therapeutic strategies is urgently needed.

Anthraquinone is initially extracted from several kinds of plants, for example, senna, cascara, aloe, frangula, and rhubarb. It has been found that anthraquinone has a wide variety of pharmacological effects, including anti-inflammation, wound healing, analgesic, antipyretic, and anti-microbial activities [7,8,9]. Some anthraquinone drugs show inhibition capability of tumor cell proliferation, which is one of the important mechanisms of anti-tumor. CC12 (NSC749232), structurally, naphtho[2,3-f]quinoxaline-7,12-dione, is one of the tetraheterocyclic homologues base on the anthraquinone backbone that is synthesized by our team [7,10,11,12]. The structure of CC12 indicates that it may have high lipophilicity, which means the compound has the probability and ability of crossing the BBB, and the consequent GBM treatment potential.

In view of unmet and urgent clinical needs, we tested the anti-GBM capability of CC12 in vitro using two GBM cell lines, including the inhibition of tumor cell proliferation, the regulation of cell cycle, and the induction of tumor cell apoptosis. In addition, the molecular mechanisms were evaluated for further understanding of the compound. A mouse xenograft model was used for understanding the in vivo therapeutic effects and the ability of crossing BBB of CC12 in the present study.

## 2. Materials and Methods

### 2.1. Preparation of CC12 and GBM Cell Lines

**Figure 1:**
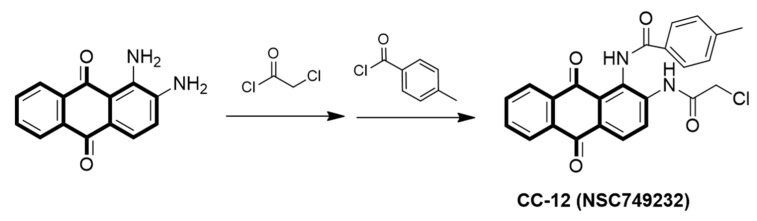
Chemical structures of CC12.

The synthesis of CC12 has been described in our earlier studies [7]; the chemical structure of CC12 is shown in Figure 1. Two human GBM cell lines were used in this study, including U87MG, which was purchased from the Bioresource Collection Research Center in Hsinchu, Taiwan; U118MG was kindly provided by Dr. Dueng-Yuan Hueng, National Defense Medical Center, Taipei, Taiwan. Both U118MG and U87MG cells were maintained in Dulbecco’s modified Eagle’s medium (DMEM), which were supplemented with 10% fetal bovine serum (FBS); both were purchased from Atlanta Biologicals (Atlanta, GA, USA) with 1% penicillin and 1% streptomycin (both, GIBCO/BRL, Grand Island, NY, USA). Fresh medium was changed every 24 h and cells were incubated in 5% CO_2_ at 37 °C.

### 2.2. MTT Assay

The 4 × 10^4^ cells were plated in 24-well plates and incubated at 37 °C for 24 h, and then CC12 was added into the medium with 2.5, 5, 10, 15, 20 μM for 24, 48, or 72 h. After treatment, 500 μL MTT solution (5 mg/mL in phosphate-buffered saline) was added into each well, the cells were continuously incubated for additional 4 h. The formazan crystals were solubilized with 500 μL DMSO, and the optical density values were detected at wavelength 570 nm by Synergy HT multi-detection micro plate reader (Awareness Technology, Palm City, FL, USA). All experiments were performed in triplicate, and the relative cell growth was expressed as a percentage of the untreated control cells (set to 100%) to allow for unwanted source of variation.

### 2.3. Western Blot Analysis

Cells were lysed in ice-cold Radioimmunoprecipitation assay, (RIPA) buffer (25mM Tris-HCl, pH 7.6, 150 mM NaCl, 1% TritonX-100, 1% sodium deoxycholate and 1% SDS) containing protease and phosphatase inhibitors (GeneTex, Irvine, CA, USA). Moreover, 100 μg protein samples were load in each lane and electrophoresed on a 5%, 10%, or 12% SDS polyacrylamide gel for separating proteins, sizes ranged: >300 kDa, 40–300 kDa, or <40 kDa, respectively. After electrophoresis, the proteins were transferred to a 0.45 μm pore size hydrophobic immobilon-P polyvinylidene fluoride (PVDF) membrane (Millipore, Bedford, MA, USA). Membranes were blocked with blocking buffer (GeneStar, Beijing, China) at room temperature for 5 min and incubated overnight at 4 °C with a 1:1000 dilution of primary antibodies, including Ki-67, phosphorylated H2A histone family member X (H2AX)S139, cyclinD1, cyclin-dependent kinase (CDK)2, CDK4, Bax, Bcl2, decoy receptor 3 (DcR3), Fas ligand (FasL), BH3 interacting-domain death agonist (Bid), poly (ADP-ribose) polymerase (PARP), caspase-3, cleaved caspase-8, cleaved caspase-9, vinculin, P53, ataxia telangiectasia and Rad3-related (ATR), and α-tubulin. After washing, the strips were incubated with a 1:10000 dilution of IR dye-conjugated anti-rabbit Immunoglobulin G (IgG) antibodies (LI-COR Biosciences, Lincoln, NE, USA) in dark room for 1h. The fluorescence density of each band on the PVDF membrane was quantified by densitometry using Odyssey^®^ CLx Infrared Imaging System (LI-COR Biosciences, Lincoln, NE, USA), taking the density of the control sample as 100%, and expressing the density of the test sample relative to the expression of the internal control as a relative value.

### 2.4. Flow Cytometry-Based Apoptosisdetections

A total of 2×10^5^ cells per well were seeded in 6-well plates and incubated for 24h, then CC12 was added. After the incubation with CC12 for 24 h or 48 h, cells were suspected with 0.05% trypsin and gathered, fixed with 75% ethanol at −20°C overnight, washed with phosphate buffered saline, and stained with 500 μL propidium iodide/RNase staining solution (BD Biosciences, Franklin Lakes, NJ, USA) for 15 min at room temperature in the dark. The 10^4^ stained cells were analyzed using FACSVerse™ laser flow cytometric analysis system (BD Biosciences) for each sample. Each experiment was done, at least, quantic. Annexin V-PE Dead Cell Apoptosis kit (BD Biosciences) was utilized for apoptotic cell death analysis, according to the manufacturer’s protocol. Cells were trypsinized and washed twice with phosphate-buffer saline, then resuspended in 100 μL Binding Buffer. 5 µL Annexin V-PE and 10 µL 7-AAD then incubated for 15 min in the dark at room temperature. Next, 400 µL binding buffer was added to the cells and 10^4^ events were acquired for each sample. The 7-AAD was analyzed at 488 nm and Annexin V-PE fluorescence was detected at 617 nm in a flow cytometer (FACSVerse™; BD Biosciences). An APO-DIRECTTM Kit (BD Biosciences) and a Flow Cytometry Mitochondrial Membrane Potential Detection Kit (BD Biosciences) were utilized for detecting DNA damage and the apoptotic cells. Each experiment was done, at least, in triplicate. Data were analyzed using FlowJo v7.6.5 software (Tree Star Inc., Ashland, OR, USA).

### 2.5. Mouse Xenograft Model and Positron Emission Tomography (PET) Scan Analysis

All protocols were authorized by the Institutional Animal Care and Use Committee of the National Defense Medical Center, Taipei, Taiwan, which is certified by the Association for Assessment and Accreditation of Laboratory Animal Care International (AAALAC 2007). The 8-week-female BALB/cAnN.Cg-Foxn1nu/CrlNarl mice, weighing 20 to 22 g, were purchased from the National Laboratory Animal Center (Taipei, Taiwan). Mice were anesthetized with isoflurane and inoculated with 1 × 10^6^ U118MG cells, subcutaneously and continuously housed until the inoculated tumor cells grew up to 50 mm^3^, and started CC12 treatment via intraperitoneal injection of 5 mg/kg/day. An equal amount of DMSO was administered on the mice of the control group. Six mice were allocated into each treatment group. [^18^F]-2-deoxy-2-fluoro-D-glucose ([^18^F]-FDG)-PET static scanning was performed after fasting overnight on Day 0, 7, 14, 21, and 28 of CC12 treatment. Mice were intraperitoneal injected with 285-295 μCi (9.5∼10.5 MBq) of [^18^F]-FDG, allowed the distribution of the [^18^F]-FDG injection for 15 min. Mice were anesthetized, imaged 20 min using animal-PET statistic scanning with BIOPET105, setting the energy window at 250–700 keV (Bioscan, Inc., Washington DC, USA). The three-dimensional (3D)-ordered-subsets expectation maximization (3D-OSEM) was used for image reconstruction; the AMIDE software, Free Software Foundation (v1.0.4) was used for image data analysis. Tumor volume was quantitated by estimating the standard uptake value (SUV) of [^18^F]-FDG, which indicates the levels of the [^18^F]-FDG in a volume of interest representative of a focal tumor (VOI) relative to the average [^18^F]-FDG levels in the whole body. The experiment lasted for 28 days, then, all mice were euthanized, and the tumor tissue was collected, weighed, and fixed by 4% paraformaldehyde on Day 29.

### 2.6. Data Analysis

All experiments were performed at least three times, and the values were reported as means ± SD (standard deviation). Differences between groups were evaluated using the Kruskal–Wallis test, followed by post hoc comparisons using the GraphPad Prime5.0 software (GraphPad Software, San Diego, CA). Statistical significance was set at *p* < 0.05.

## 3. Results

### 3.1. CC12 Induced Tumor Cell Death and Inhibited Tumor Cell Proliferation

**Figure 2:**
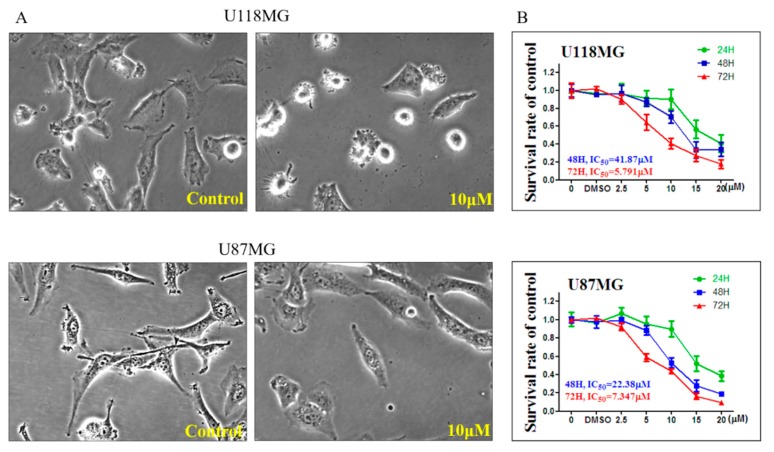
CC12 induced cell death and decreased the survival rate with time- and dose-dependent manners in glioblastoma (GBM) cell lines. (**A**) GBM cells showed shrinking and low cell density after CC12 treatment for 24 h. (**B**) (3-(4,5-Dimethylthiazol-2-yl)-2,5-diphenyltetrazolium bromide (MTT)assay results indicated the decease of the survival rate in both GBM cell lines.

After treated with 10 μM CC12 for 24 h, both U118MG and U87MG cells were shrinking, meaning that CC12 may induce tumor cell death (Figure 2A). This effect was time- and dose- dependent; results of MTT assay showed that the survival rates decreased when the CC12 concentrations or the treatment time increased. Both GBM cell lines showed this effect, in the U118MG cell line, when treated with CC12 for 48 h and 72 h; the IC50 were 41.87 and 5.791 μM, respectively. In the U87MG cell line, when treated with CC12 for 48 h and 72 h, the IC50 was 22.38 and 7.347 μM, respectively (Figure 2B).

**Figure 3:**
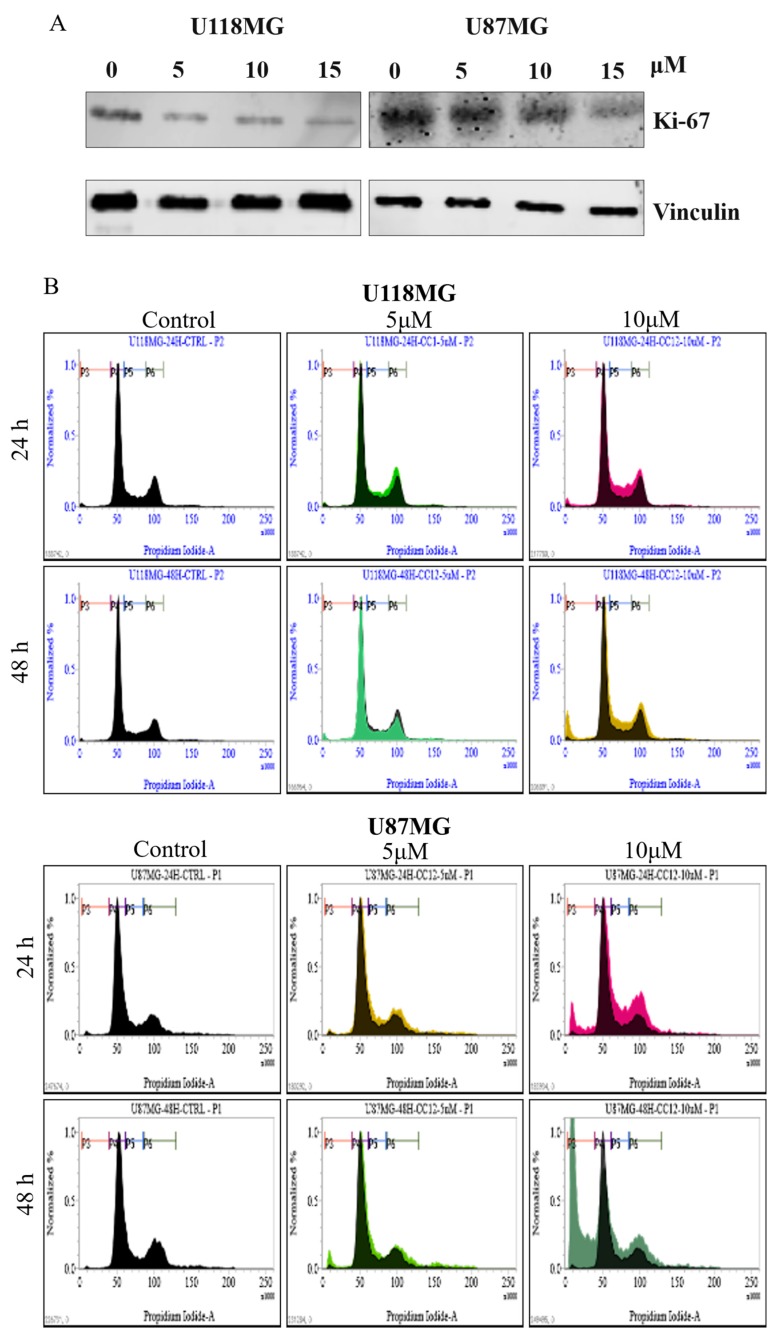

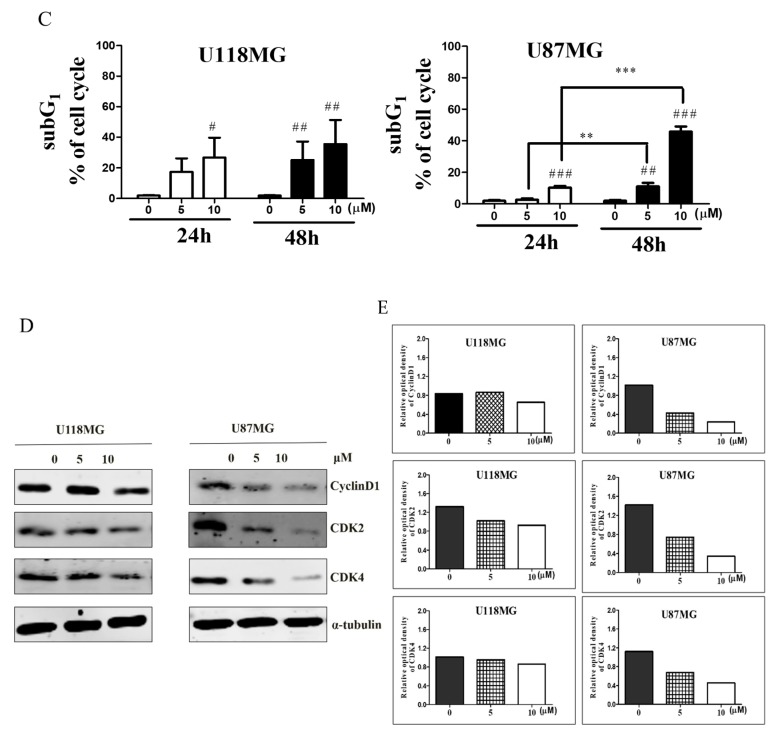
CC12 inhibited cell proliferation. (**A**) Western blot results showed the decrease of the proliferation marker Ki-67 after CC12 treatment. Flow cytometric analysis indicated that CC12 treatment increased the apoptosis by (**B**) the presence of hypodiploid peak and (**C**) the significant increase of the proportions of sub-G1 phase cells. (**D**) The protein expressions of G1 phase drivers decreased in U87MG cells; however, there were no changes in U118MG cells. (**E**) The quantification analysis of the western blot results. # and ## indicates significant difference compared with the control group, *p* < 0.05 and < 0.01, respectively; * and *** indicates the significant difference between 24 h and 48 h treatments within the same concentration groups, *p* < 0.05 and < 0.01, respectively.

CC12 also inhibited tumor cell proliferation, represented by both proliferation marker Ki-67 expression and the results of flow cytometric analysis. The expression of Ki-67 reduced after CC12 treatment, and the reduction levels increased with higher CC12 concentrations (Figure 3A). The hypodiploid peaks were shown in the histograms of flow cytometry, meaning the increases in the proportion of apoptotic cells (Figure 3B), and the proportions of sub-G1 phase cells also increased significantly after CC12 treatment, with 2.6% and 10.3% at 24 h, and 11.1% and 45.8% at 48 h after treatment, with 5 and 10 μM CC12, respectively (Figure 3C). Western blot of the G1 phase drivers were performed to evaluate whether these proteins were involved in the regulation of cell cycle. The results showed that in both U118MG and U87MG cell lines, cyclinD1 decreased with the increased concentration of CC12. Further, the expressions of CDK2 and CDK4 decreased in U87MG cells; however, there was no change in U118MG cells (Figure 3D).

### 3.2. CC12 Induced Apoptosis of Tumor Cells

**Figure 4:**
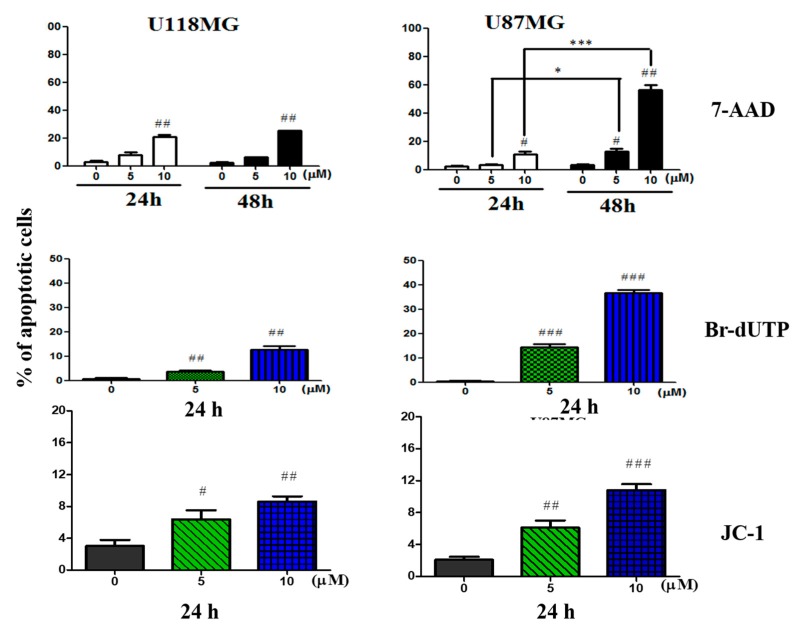
CC12 treatment increased the apoptosis rate and DNA damage. Results of 7-AAD, Br-dUTP, and JC-1 staining all indicated that the apoptotic cells were more after GBM cells were treated by CC12. # and ## indicates significant difference compared with the control group, *p* < 0.05 and <0.01, respectively; * and *** indicates significant difference between 24 h and 48 h treatments within the same concentration groups, *p* < 0.05 and <0.01, respectively.

We used 7-AAD, Br-dUTP, and JC-1 staining to evaluate whether the cell death included by CC12 was through apoptosis. The increased annexin V-PE positive and 7-AAD negative cells, or increased cells in the A4 area, indicated the occurrence of apoptosis were shown in both U118MG and U87MG cell lines after treatment with different concentrations of CC12 (data not shown). The apoptosis proportion, after treatment, with 10 μM CC12 for 24 h in U118MG cells was 9.5%, as well as 13.2% in U87MG cells. In Br-dUTP staining, the apoptotic cells of the U118MG cell line significantly increased to 4.5% and 13.4% after treatment of 5 and 10 μM CC12 for 24 h, respectively. Moreover, the proportion of U87MG cells with DNA fragmentation content increased from 11.5% to 34.2%. JC-1 staining also showed similar results, the apoptotic cells increased in both bU118MG and U87MG cell lines (Figure 4).

**Figure 5:**
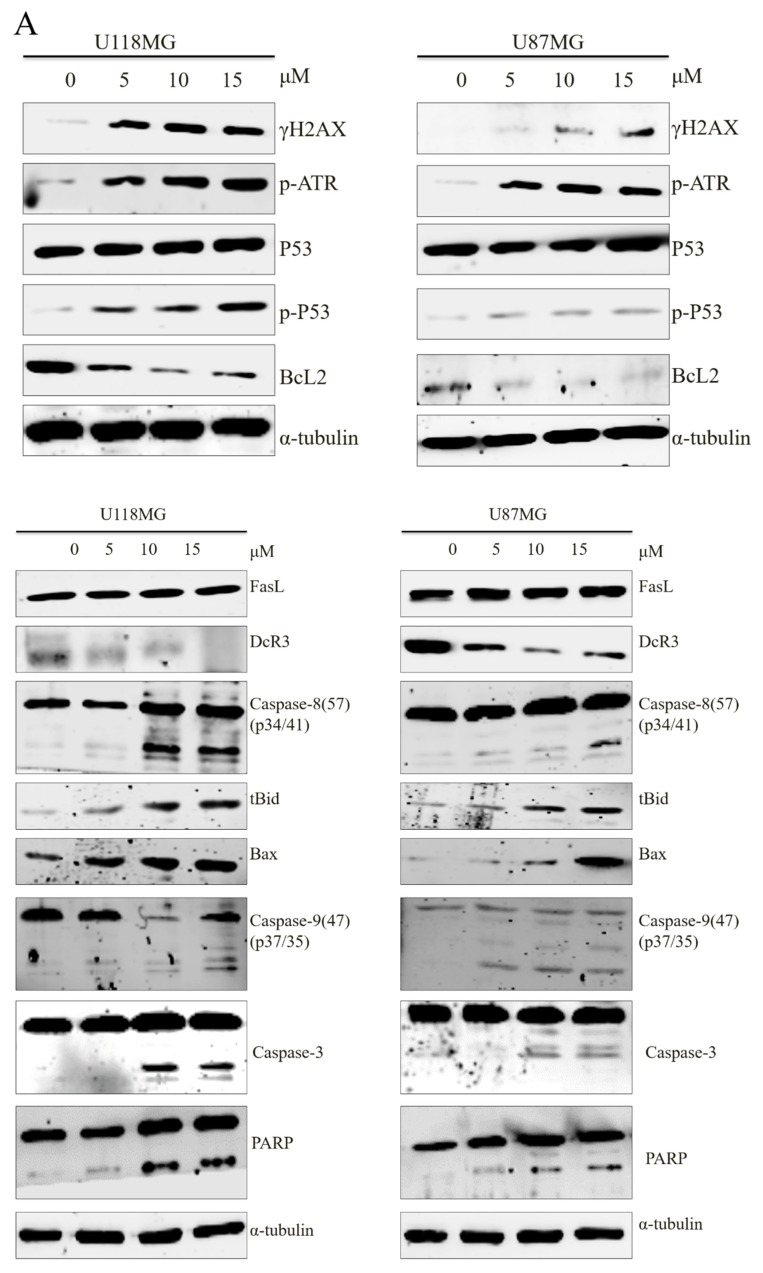

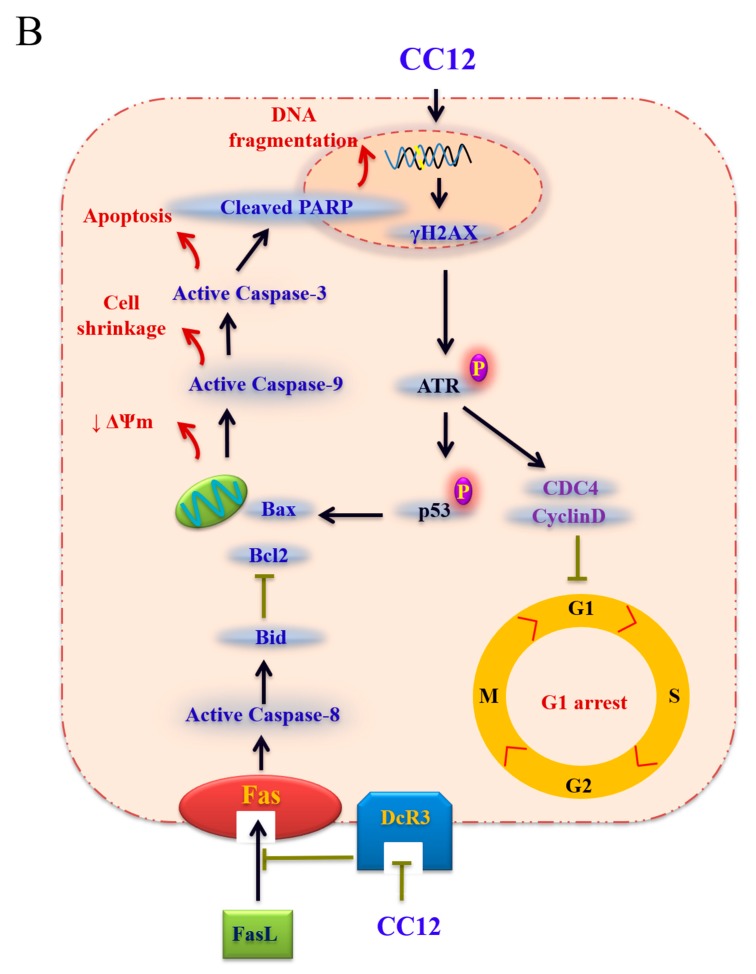
CC12 affected the expression of the proteins of intrinsic and extrinsic apoptotic pathways, and tumor prognostic marker Decoy receptor 3 (DcR3). (**A**) Western blot. (**B**) The possible mechanism.

Both intrinsic and extrinsic apoptotic pathway were evaluated for the mechanism of CC12-associated tumor cell apoptosis by Western blot (Figure 5A). γH2AX is a marker of DNA damage, which is one of the major changes when apoptosis. The expression of γH2AX was induced by CC12 treatment. ATR, P53, and phosphor-P53, Bid, Bax, caspase-3, phosphorylated caspase-9, caspase-8, and PARP increased after CC12 treatment and were dose-dependent in both U118MG and U87MG cell lines; Bcl2 expression reduced after CC12 treatment. No change was found in FasL levels. As a tumor prognostic marker, we found that DcR3 expressed in GBM, like other malignancies, and the amount decreased after CC12 treatment and with dose-dependent manner in both GBM cell lines. A proposed mechanism base on the results is shown in Figure 5B.

### 3.3. CC12 Showed Tumor Elimination Effects In Vivo

**Figure 6:**
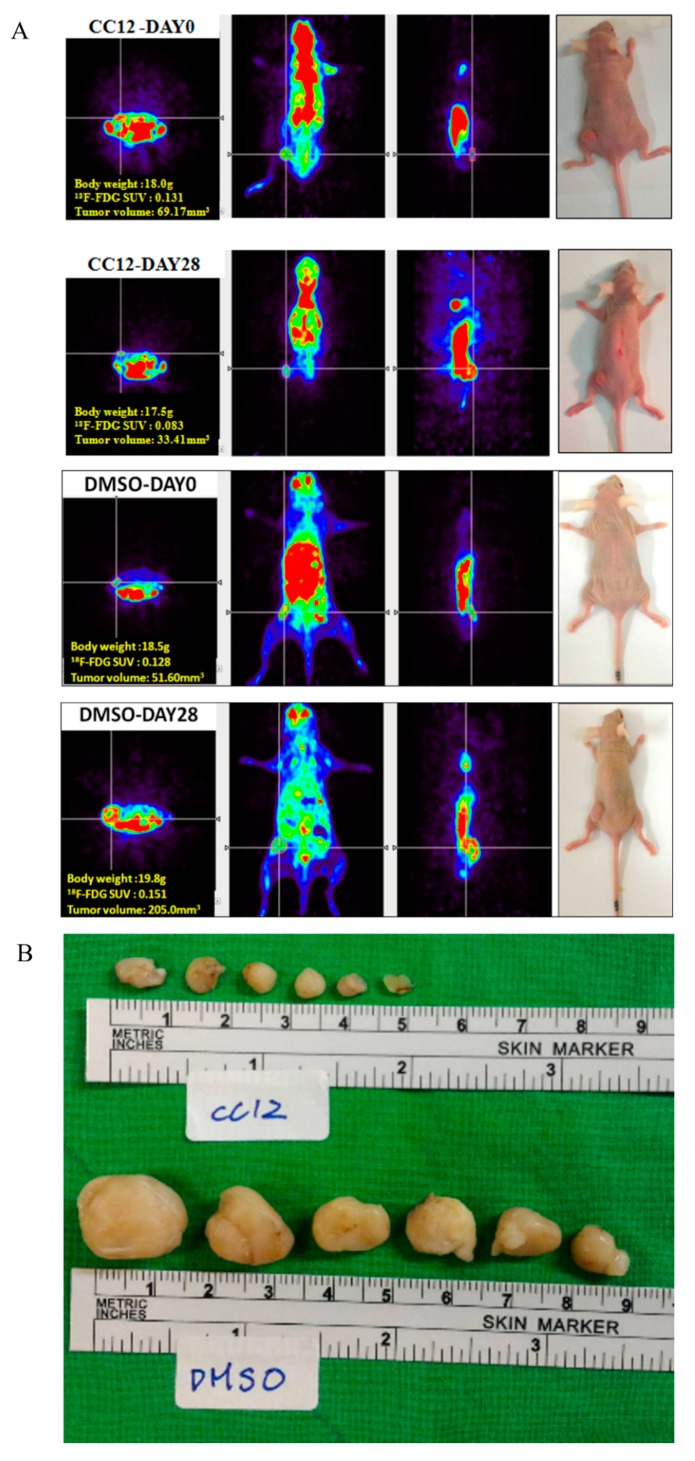

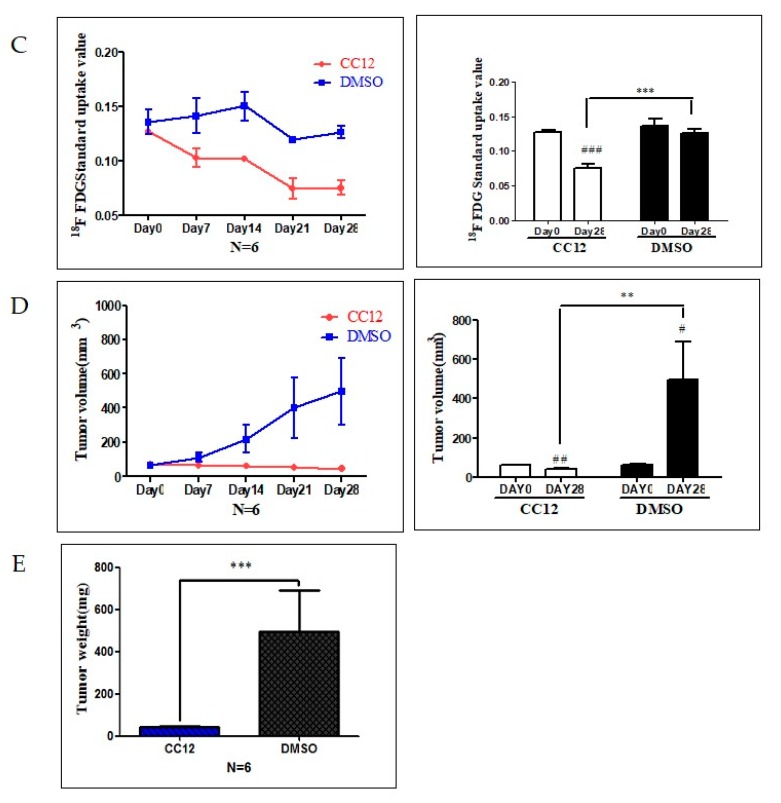
CC12 inhibited the growth of the U118MG in the mouse xenograft model. (**A**) Positron emission tomography with 2-deoxy-2-[fluorine-18] fluoro- D-glucose([^18^F]-FDG PET) image of Day 0 and 28 of each treatment group. (**B**) Images of the tumor at the end of each treatment group. (**C**) [^18^F]-FDG PET image indicated that the uptake values reduced significantly from Day 0 to Day 28 after CC12 treatment, while in mice of the DMSO group, no significant change was found. (**D**) The tumor volumes continuously increased in mice of the DMSO group; however, no significant change was found in those of CC12 treatment group. On Day 28, the difference between the CC12 and DMSO groups were significant. (**E**) The tumor weight at the end of the in vivo study showed the significant difference between the CC12 treatment group and the DMSO control group. Data are presented as mean ± SD, statistically signification values of ** *p* < 0.01, *** *p* < 0.001 were appraised compared with the control, # *p* < 0.05, ## *p* < 0.01, ### *p* < 0.001 comparing days 0 and 28. Significantly different from control; ANOVA with Tukey’s test.

Further, we used a xenograft mouse model to evaluate the potential therapeutic effects of CC12 in vivo. On Day 0, the mean uptake value of [^18^F]-FDG in CC12 group was 0.139 ± 0.02, consistent with those in the DMSO group, as 0.136 ± 0.0, (*p* > 0.05), while those in the CC12 group were significantly lower than the DMSO group on Day 28, with the mean values as 0.097 ± 0.02 vs. 0.138 ± 0.01 (*p* < 0.01). Furthermore, the tumor volumes reduced in mice of the CC12 group; in contrast, the tumor volume enlarged in mice of the DMSO group. On Day 0, mean tumor volume of the CC12 group was 61.15 ± 6.89 mm^3^, similar to that in the DMSO group, as 64.01 ± 14.08 mm^3^ (*p* > 0.05). On Day 28, the tumor volumes were significantly different between the two treatment groups, as 44 ± 12 mm^3^ in the CC12 group and 496 ± 480 mm^3^ in the DMSO group (*p* < 0.05). At the end of the experiment (Day 29), the tumors were isolated after the mice were sacrificed. The tumors were significantly shrunken in the mice of the CC12 group, compared to that in the DMSO group, with the tumor weight 43.6 ± 12. mg and 548 ± 554 mg in CC12 and DMSO groups, respectively (*p* < 0.01) (Figure 6).

**Figure 7:**
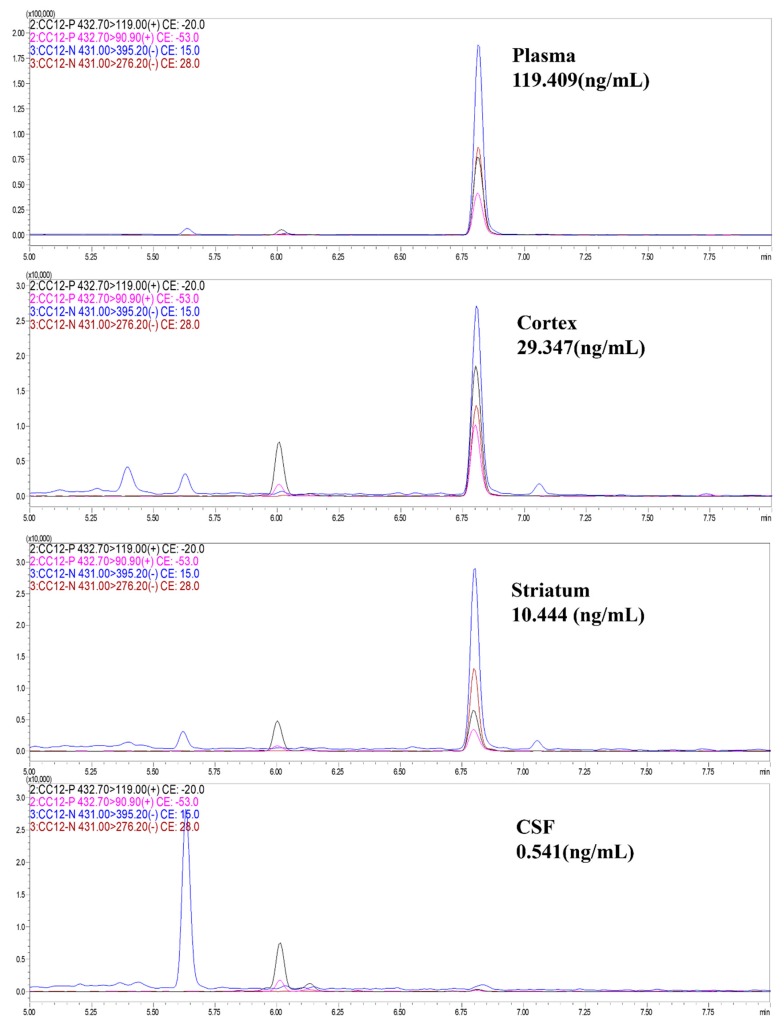
CC12 can cross the brain-blood barrier. HPLC/MS analysis results indicated that CC12 can be detected in plasma, cortex, striatum, and CSF from mice that received 5 mg/kg CC12 for 28 days.

Since GBM is the major type of brain malignancies, the effective treatment should have ability to cross BBB. We measured the concentration of CC12 in different sources of the samples from mice received 5 mg/kg CC12 for 28 days. CC12 can be detected in the CNS by the HPLC/MS method, including cortex, striatum, and CSF, indicating that CC12 can diffuse into the brain, then induce the apoptosis of the brain tumor (Figure 7).

## 4. Discussion

In both U118MG and U87MG GBM cell lines, CC12 incubation reduced the cell survival rate, and the level of reduction increased by treatment time and CC12 dose. The tumor cell proliferation was inhibited, represented by decreased Ki-67 expression and increased sub-G1 phase cells, significantly. CC12 also increased the apoptosis rate observed by flow cytometry-base analysis. For the mechanism of cell cycle, apoptosis, and tumor prognosis, we found that G1 phase drivers decreased after CC12 treatment in U87MG cells, including cyclinD1, CDK2, and CDK4; apoptotic proteins γ-H2AX, Bid, Bax, caspase 3,8 and 9, PARP, P53, and ATR increased, and the anti-apoptotic protein Bcl2 decreased in U118MG and U87MG cells after CC12 treatment. Furthermore, CC12 can induced the decrease of tumor prognostic marker DcR3. From the results of the in vivo experiments, we observed the effect of tumor size reduction of CC12; in addition, we confirmed that CC12 could cross the BBB by HPLC analysis.

Cell death pathways are frequently inactivated in human cancers, including GBM. For development of therapeutic strategy, re-activate the normal cell death pathway is a promising target. As well as oncogenesis, inactivated cell death signaling is the major source of treatment resistance; thus, we tested the apoptosis-regulation capability of the synthesized compound CC12 on GBM cell lines, and both intrinsic and extrinsic apoptotic pathways were evaluated. In GBM, high expression levels of Bcl2 proteins expression in several GBM cell lines have been reported, indicating that Bcl2 with a partial caspase-dependent mechanism is important in the immortalization process of GBM [13]. Many GBM drugs have been developed to target the Bcl2 pathway, for example, ABT-737 inhibits both Bcl2 and Bcl-XL, and can induce apoptosis of GBM in vitro and in vivo [14]. The downstream proteins Bid was accumulated, Bax was activated, and then led to caspase-dependent apoptosis [14,15]. CC12 may—through this pathway—induce GBM cell apoptosis.

However, we did not find any change in the expression level of FasL after CC12 treatment, neither in U118MG nor in U87MG cells. Because FasL is the starter of the extrinsic apoptotic pathways, CC12′s anti-tumor effects probably via another mechanism. The downstream molecules of FasL we evaluated in the present study included caspase 3 and PARP. Caspase 3 can also be regulated by intrinsic apoptotic proteins, including Bid [15], so the changes of caspase 3 after CC12 treatment were caused by the alteration of the intrinsic apoptotic signal pathway. Studies of the mechanisms of treatment and resistance of temozolomide, the most frequently used GBM drug, indicated that PARP can improve the sensitivity to temozolomide in malignances, including GBM [16]. PARP is a DNA damage sensor of single strand breaks and participates in single strand break repair. The increase of PARP after CC12 treatment was consistent with our results of Br-dUTP staining, indicating the DNA fragmentation, and the higher levels of γH2AX expression induced by CC12, a symbol of DNA damage. However, not only DNA damage, the association of γH2AX and cell cycle arrest at the G1 phase was reported in GBM cells [17], which were seen in our results, too. 

DcR3 is a soluble receptor belonging to the tumor necrosis factor receptor superfamily that binds competitively to FasL [18]. High level expression of DcR3 is associated with poor prognosis of many different malignancies, including GBM [19]. CC12 can reduce the expression of DcR3; however, the mechanism may be FasL independent because of the ‘no changed’ level after CC12 treatment, which we mentioned above. DcR3 can bind to other members of the tumor necrosis factor receptor superfamily, including LIGHT and TNF-like molecule 1A [20,21]; the actual binding molecules in GBM after CC12 treatment need further investigation. The oncogenesis mechanism of DcR3 have been evaluated in a mouse tumor model, indicating that DcR3 regulated macrophage differentiation and contributed to tumor progression [22]. The role of immune moderation and inflammation of tumor initiation and progression via DcR3 in GBM can be evaluated for developing new treatment targets.

In conclusion, a newly synthesized compound CC12 was investigated for the potential therapy for GBM; the results indicated that CC12 may have anti-tumor ability through the regulation of cell cycle and apoptosis, in vitro and in vivo, and has the ability to cross BBB.
